# *JAK2* V617F-Dependent Upregulation of PU.1 Expression in the Peripheral Blood of Myeloproliferative Neoplasm Patients

**DOI:** 10.1371/journal.pone.0022148

**Published:** 2011-07-18

**Authors:** Tamotsu Irino, Munehiro Uemura, Humitsugu Yamane, Shigeto Umemura, Takahiko Utsumi, Naoki Kakazu, Taku Shirakawa, Mitsuhiro Ito, Takayo Suzuki, Kazuo Kinoshita

**Affiliations:** 1 Shiga Medical Center for Adults, Moriyama, Japan; 2 Division of Medical Biophysics, Kobe University Graduate School of Health Science, Kobe, Japan; 3 Shiga Medical Center Research Institute, Moriyama, Japan; 4 Department of Environmental and Preventive Medicine, Shimane University School of Medicine, Izumo, Japan; University of Bergen, Norway

## Abstract

Myeloproliferative neoplasms (MPN) are multiple disease entities characterized by clonal expansion of one or more of the myeloid lineages (i.e. granulocytic, erythroid, megakaryocytic and mast cell). *JAK2* mutations, such as the common V617F substitution and the less common exon 12 mutations, are frequently detected in such tumor cells and have been incorporated into the diagnostic criteria published by the World Health Organization since 2008. However, the mechanism by which these mutations contribute to MPN development is poorly understood. We examined gene expression profiles of MPN patients focusing on genes in the JAK–STAT signaling pathway using low-density real-time PCR arrays. We identified the following 2 upregulated genes in MPN patients: a known target of the JAK–STAT axis, *SOCS3*, and a potentially novel target, *SPI1*, encoding PU.1. Induction of PU.1 expression by *JAK2* V617F in *JAK2*-wildtype K562 cells and its downregulation by *JAK2* siRNA transfection in *JAK2* V617F-positive HEL cells supported this possibility. We also found that the ABL1 kinase inhibitor imatinib was very effective in suppressing PU.1 expression in BCR-ABL1-positive K562 cells but not in HEL cells. This suggests that PU.1 expression is regulated by both JAK2 and ABL1. The contribution of the two kinases in driving PU.1 expression was dominant for JAK2 and ABL1 in HEL and K562 cells, respectively. Therefore, PU.1 may be a common transcription factor upregulated in MPN. PU.1 is a transcription factor required for myeloid differentiation and is implicated in erythroid leukemia. Therefore, expression of PU.1 downstream of activated JAK2 may explain why *JAK2* mutations are frequently observed in MPN patients.

## Introduction

Genetic testing to diagnose cancer is now becoming a common clinical practice. This trend is quite reasonable from the viewpoint that cancer is a genetic disease. In 2008, the World Health Organization incorporated genetic tests for the gene of Janus kinase 2 (*JAK2*) V617F mutation and other functionally similar mutations (such as *JAK2* exon 12 mutations) into the diagnostic criteria for myeloproliferative neoplasms (MPN), a collection of hematological malignancies that include polycythemia vera (PV), essential thrombocythemia (ET), and primary myelofibrosis [1]. It has been reported that PV patients possess a homozygous *JAK2* V617F mutation, while a heterozygous mutation is common in ET patients [2]. Although such correlations indicate a dose effect of these mutations on disease manifestation, the molecular mechanism of this phenomenon remains unclear.

JAK kinases are cytoplasmic molecules that transmit signals from cytokine receptors to signal transducer and activator of transcription (STAT) transcription factors [3]–[5]. Since *JAK2* mutations observed in MPN patients are known to exert their effects by activating downstream signaling pathways leading to the activation of target genes, it is possible that the effects of all types of mutations observed in *JAK2* and other functionally related genes, such as myeloproliferative leukemia virus oncogene (*MPL*), funnel into the activation of a common set of genes. If so, determination of the mRNA abundance of such genes may be a substitute for the laborious screening of possible genetic mutations.

In this study, we examined the expression profiles of 84 JAK–STAT-related genes in peripheral blood samples taken from 26 MPN patients to determine molecular signatures of the activated JAK–STAT signaling pathway. We found that the suppressor of cytokine signaling 3 (*SOCS3*) expression was significantly elevated in MPN patients with a *JAK2* V617F mutation, suggesting that *SOCS3* mRNA in peripheral blood can be used as a biomarker for diagnosis and assessment of MPN patients. In addition, we report here a novel link between JAK2 and a hematopoietic transcription factor, PU.1, which was verified using *in vitro* cell culture experiments. As PU.1 is a regulator of proliferation and differentiation of erythroid, myeloid, and lymphoid cells [6], the effect of *JAK2* mutations may be mediated partly through upregulation of PU.1. In addition, through pharmacological inhibition of *c-abl* oncogene 1 (ABL1) kinase, both PU.1 and SOCS3 appeared to be regulated with a breakpoint cluster region (BCR)-ABL1 fusion protein in K562 cells. In contrast to *JAK2* V617F-positive HEL cells, PU.1 and SOCS3 expression in K562 cells were JAK2-independent, suggesting that PU.1 and SOCS3 may be a common downstream target of oncogenic JAK2 and ABL1 signaling.

## Materials and Methods

### Patients and Samples

Twenty-six patients diagnosed with MPN at Shiga Medical Center for Adults in 2008 and 2009 and 11 healthy volunteers were enrolled in this study (Supplementary Table S1). Genomic DNA and total RNA were isolated from their peripheral blood using a QuickGene DNA whole blood kit S (DB-S, Fuji Film, Tokyo, Japan) and a RiboPure-Blood kit with RNAlater solution (Applied Biosystems, Foster City, CA), respectively. Integrity of DNA and RNA was verified by agarose gel electrophoresis and capillary electrophoresis using Experion (Bio-Rad, Hercules, CA), respectively. Written informed consent was obtained from all participants, and samples were collected and analyzed. All procedures were approved by the Ethical Committee of Shiga Medical Center for Adults.

### Mutation Analysis

The presence of a *JAK2* V617F mutation was determined by the following 3 methods: allele-specific PCR, quantitative allele-specific PCR, and direct sequencing. Allele-specific PCR was performed as reported previously [7]. Samples with a mutant-specific band stronger than that of a mixture of genomic DNA from HEL (V617F-type *JAK2*) and K562 (wild-type *JAK2*) cell lines at a ratio of 1∶99 was determined to be positive for a JAK2 mutation.

Quantitative allele-specific PCR was performed in triplicate in 18-µl reactions containing 1× SYBR Premix Ex Taq II (Takara, Otsu, Japan), 500 nM forward primer, 500 nM reverse primer, and 36 ng genomic DNA using an Mx3000P real-time thermal cycler (Agilent, Santa Clara, CA). The PCR conditions were as follows: 95°C for 30 s, 40 cycles at 95°C for 15 s, and then 58°C for 1 min, followed by a segment for dissociation curve analysis. The wild-type and V617F alleles were amplified using the following primer pairs: JAK2-ASPCR-F3 and JAK2-ASPCR-R2 for the wild-type allele, and JAK2-ASPCR-F4 and JAK2-ASPCR-R2 for the V617F allele. Primer sequences are listed in Supplementary Table S2. Standard curves were drawn using defined copy numbers (7.2×10^4^, 7.2×10^3^, 7.2×10^2^, and 7.2×10^1^) of PCR products prepared using primers JAK2-Frag-1F and JAK2-Frag-1R for genomic DNA from HEL (template for the V617F allele) and K562 (template for the wild-type *JAK2* allele) cells. The V617F mutation burden was calculated as the ratio of the amount of V617F DNA to the sum of V617F and wild-type DNA. The validity of this assay was confirmed by comparing these results with those obtained using a commercially available kit (JAK2 MutaQuant Kit, Ipsogen, New Haven, CT).

Direct sequencing was performed by sequencing the 364-bp band by allele-specific PCR [7] from the primer JAK2-Seq-1R using BigDye Terminator v1.1 and BigDye XTerminator with an ABI PRISM 3130 sequencer (Applied Biosystems). Samples that displayed clear upward T peaks at the wild-type G position (the first position of codon 617) were determined to be V617F positive.

*JAK2* exon 12 and *MPL* exon 10 mutations were also determined by direct sequencing as described [8], [9].

### Quantitative PCR Assay

First-strand cDNA was synthesized using an iScript kit (Bio-Rad) from peripheral blood total RNA and a PrimeScript RT reagent kit with gDNA Eraser (Takara) from cell line RNA. Expression profiles of JAK–STAT-related genes were analyzed with a PCR array PHAS-039 (SABiosciences, Frederick, MD) and RT2 Real-Time SYBR Green/ROX PCR master mix using an Mx3000P cycler (Agilent). Statistical analysis of obtained Ct values was performed using web-based software (RT2 Profiler) provided by SABiosciences. Data obtained from 37 plates are listed in Supplementary Table S3.

Quantitation of *SOCS3*, *SOCS1*, *SPI1*, hypoxanthine phosphoribosyl-transferase 1 (*HPRT1*), and ribosomal *18S* RNA was performed in triplicate using SYBR Premix Ex Taq II (Takara) for patient samples and SYBR Premix DimerEraser (Takara) for cell line samples. The primer sequences for *SOCS1* and *SOCS3* were as reported previously [10]. The primers for *SPI1* (HA102872-F and HA102872-R), *HPRT1* (HA067805-F and HA067805-R), and *18S* (HA067799-F and HA067799-R) were purchased (Takara). Standard curves for each gene were drawn by measuring 10-fold serial dilutions of cDNA samples derived from the RNA of HEL cells. The mean values of measurements were normalized by the amounts of *HPRT1* and *18S* for patient and cell line samples, respectively. When we first attempted a calibration using *GAPDH* mRNA, we found a 2-fold reduction of *GAPDH* in V617F-expressing K562 cells compared with the mock transfectant (data not shown). Therefore, we started using the more stable *HPRT1* for peripheral blood studies and *18S* ribosomal RNA for cell line studies. Statistical analyses were performed using Prism 4 software (Graph Pad, San Diego, CA).

### Vector Construction

Coding sequences for wild-type and V617F-type *JAK2* were amplified from cDNA obtained from K562 and HEL cells, respectively, using primers hJAK2-F-1F and hJAK2-F-1R. Fragments obtained after digestion with *Sal*I were inserted at the *Xho*I site of pFB-da-Puro, a modified retrovirus vector of pFB (Agilent), by insertion of an internal ribosomal entry site and a puromycin resistance gene. The obtained plasmids for the wild-type and V617F-type JAK2 retroviral vectors were designated phJAK2-FBP and phJAK2V617F-FBP, respectively.

### Cell Culture

K562 and HEL cell lines were obtained from the Japanese Collection of Research Bioresources (Osaka, Japan) and cultured in RPMI1640 medium with 10% fetal bovine serum and penicillin/streptomycin (Invitrogen, Carlsbad, CA).

phJAK2-FBP, phJAK2V617F-FBP, or pFB-da-Puro were cotransfected using FuGENE6 (Roche, Mannheim, Germany) into HEK293T cells with pCL-Ampho (Imgenex, San Diego, CA) to prepare a culture supernatant containing a JAK2 retrovirus, which was used to infect K562 and HEL cells by spinfection. After culturing in the presence of puromycin (0.6 µg/ml for K562 and 1.3 µg/ml for HEL) for 10–20 days, cells were lysed for RNA extraction using a QuickGene RNA cultured cell kit S (RC-S, Fuji Film).

Three types of siRNA for the human *JAK2* gene and 2 negative control siRNAs were purchased (Silencer Select siRNAs, Applied Biosystems, s7649 as siRNA1, s7650 as siRNA2, s7651 as siRNA3, negative control #1 as NC1, and negative control #2 as NC2). Samples of 30 pmol of these siRNAs were transfected into 2×10^6^ HEL and K562 cells, using Nucleofector II (Amaxa, Köln, Germany) with a Cell Line Nucleofector Kit V (Amaxa) and a Program X-005. Then, cells were transferred to 6-well plates containing 4 ml of culture medium and maintained at 37°C under a 5% CO_2_ atmosphere until protein analysis on day 1 and RNA preparation on day 2.

JAK2-specific inhibitor AG490 (Merck, Darmstadt, Germany) and ABL1 inhibitor imatinib (Toronto Research Chemicals, North York, Canada) were purchased and dissolved as stocks at 10 mM with dimethyl sulfoxide and 10 mM with phosphate-buffered saline, respectively. The vehicle concentrations were the same for all culture conditions. Cells were harvested on day 1 for JAK2 phosphorylation analysis and on days 1 and 2 for RNA preparation.

### Western Blotting

Cells were lysed on ice with RIPA buffer (50 mM Tris-Cl, 150 mM NaCl, 1% NP-40, 0.5% deoxycholic acid, 0.1% SDS) supplemented with 1× Complete Protease Inhibitor Cocktail (Roche) and 4× Phosphatase Inhibitor Cocktail 2 (Sigma-Aldrich, St. Louis, MO). Cell lysate cleared by centrifugation was resolved by 4%–20% gradient polyacrylamide gel (Miniprotean TGX gel, Bio-rad). Proteins were semi-dry transferred to Immobilon-CF PVDF membrane (Millipore) and probed with specified antibodies using a SNAP i.d. Protein Detection System (Millipore, Billerica, MA). Bound antibodies were detected using either an infrared fluorescence scanner, Odyssey (Li-cor, Lincoln, NE) or an LAS-3000 mini CCD camera (Fuji). Quantitation of band intensity was performed using Odyssey version 1.2 (Li-cor). Rabbit monoclonal antibodies for human JAK2 (D2E12) and tyrosine 1007/1008-phosphorylated JAK2 (C80C3) were purchased from Cell Signaling (Danvers, MA). Rabbit polyclonal antibodies against human elongation factor 2 (EF-2) were purchased from Santa Cruz Biotechnology (sc-25634; Santa Cruz, CA). Alexa 680-labeled anti-rabbit IgG antibodies (Invitrogen, Carlsbad, CA) and horseradish peroxidase-labeled anti-rabbit IgG (Biosource, Camarillo, CA) were purchased. Chemiluminescence reaction was performed using ECL Plus reagent (GE Healthcare, Piscataway, NJ).

## Results

### *JAK2* V617F mutation analysis

Genomic DNA purified from peripheral blood samples from 26 patients with MPN, diagnosed at Shiga Medical Center for Adults in 2008 and 2009, was analyzed to determine *JAK2* V617F mutation status. The results of these tests are listed with clinical parameters in Supplementary Table S1. None of our patients had *JAK2* exon 12 or *MPL* mutations.

### Expression profiles of genes related to the JAK–STAT signaling pathway

Using total RNA purified from peripheral blood of 26 patients and 11 healthy volunteers, expression profiles of 84 genes related to JAK–STAT-mediated signaling were analyzed with a commercially available PCR array (SABiosciences). The Ct-value data were normalized and compared using a web-based analysis tool provided by SABiosciences. The source Ct-value data are shown in Supplementary Table S3. First, we classified our MPN patients into 3 groups based on their *JAK2* V617F mutation burden as determined by quantitative allele-specific PCR. Group 1 comprised patients with a mutation load of 50%–100%, representing most PV patients. Group 2 included patients with a mutation burden of 1%–50%, representing approximately half of the ET patients and 3 PV patients. Group 3 comprised patients with less than a 1% mutation load, representing the *JAK2* V617F-negative ET patients. The normal volunteers were allocated to a control group. The characteristics of the patient groups are listed in Table 1.

**Table 1:** Sample Category Statistics.

Category	Age	Female	Male	Mutational Burden (%)	WBC (×10^9^/L)	RBC (×10^9^/L)	Hb (g/dL)	Ht (%)	PLT (×10^9^/L)
				[0]	[3.4∼9.2]	[F 3500∼5000; M 4000∼5600]	[F 10.9∼14.7; M 13.2∼17.1]	[F 32.5∼44.7; M 38.6∼50.7]	[14.8∼35.2]
PV	63±14	6	6	64±24	16±6	6330±1131	15±2	48±6	627±270
ET	63±10	9	5	12±14	7±2	4013±646	13±1	39±4	732±259
ET+	65±11	6	2	21±13	7±1	4208±550	13±1	40±2	613±227
ET−	61±10	3	3	0±0	7±2	3753±722	13±2	37±5	891±222
Group 1	63±15	3	6	76±7	18±5	6634±1070	16±2	50±5	578±285
Group 2	65±11	9	2	22±13	8±3	4593±946	13±1	41±3	652±223
Group 3	61±10	3	3	0±0	7±2	3753±722	13±2	37±5	891±222
Control	48±8	4	7						

Mean value and standard deviation of age, mutation burden, white blood cell count, red blood cell count, hemoglobin, hematocrit, and platelet count at the time of blood sampling are shown. Numbers of females and males are shown. The normal range of each test is indicated in the brackets with female (F) or male (M)-specific ranges if available. Categories based on disease are: PV for polycythemia vera; ET for total cases of essential thrombocythemia; ET+ for V617F-positive ET, ET− for V617F-negative ET; and control for healthy volunteers. Group 1, group 2, and group 3 represents cases with a V617F mutation burden 50%–100%, 1%–50%, and 0%–1%, respectively.

We looked for differences in the gene expression profiles of patients in group 1 and the control group and found that 14 genes were upregulated more than 2-fold and 33 genes were downregulated more than 2-fold in group 1 patients compared with controls, respectively (Table 2). Of the 14 upregulated genes in group 1, 3 genes—β actin (*ACTB*), *SOCS3*, and spleen focus forming virus proviral integration oncogene 1 (*SPI1*)—were also upregulated in group 2. Therefore, these 3 genes appeared to be strongly associated with the *JAK2* V617F mutation. We focused on *SOCS3* and *SPI1* in our subsequent studies.

**Table 2:** Differential expression of genes in the JAK–STAT signaling pathway in MPN patients.

	Gene	Group 1	Group 2	Group 3
1	*ACTB*	10.9	7.1	(−3.1)
2	*SOCS3*	7.5	2.2	(−1.7)
3	*JAK2*	4.4	(2.7)	(2.0)
4	*JAK3*	4.2	(2.0)	(1.7)
5	*FAS*	4.1	(2.4)	2.7
6	*SPI1*	3.9	2.1	(1.6)
7	*STAT5B*	3.6	(1.5)	(−1.5)
8	*IFNGR1*	2.7	(1.6)	(1.1)
9	*SP1*	2.6	(1.9)	(1.3)
10	*IRF9*	2.6	(1.6)	(−1.1)
11	*STUB1*	2.4	(1.6)	(1.8)
12	*GAPDH*	2.3	(−1.2)	2.0
13	*JUNB*	2.2	(−1.1)	(−1.3)
14	*FCGR1A*	2.1	(1.3)	−2.2
15	*STAT3*	1.7	(1.5)	(1.1)
16	*SRC*	−1.6	(1.1)	(1.3)
17	*HMGA1*	−1.6	(−1.2)	(−1.4)
18	*HPRT1*	−1.8	(−1.6)	−1.5
19	*IL2RA*	−1.9	(−1.6)	−1.8
20	*CSF2RB*	−1.9	−1.6	−3.5
21	*SOCS1*	−2.2	−2.0	−3.9
22	*IL20*	−2.2	(1.0)	(−2.0)
23	*TYK2*	−2.3	(−1.4)	−2.0
24	*SOCS2*	−2.3	(−1.4)	(−2.5)
25	*EPOR*	−2.3	(−1.5)	−2.2
26	*YY1*	−2.5	−1.7	−2.8
27	*STAT1*	−2.5	(−1.5)	−2.6
28	*SOCS4*	−2.5	(−1.6)	(−2.5)
29	*MMP3*	−2.5	(−1.4)	(−2.8)
30	*IL2RG*	−2.6	−2.1	−3.4
31	*IL4*	−2.7	(−1.6)	(−2.8)
32	*SOCS5*	−2.7	(−1.6)	(−2.8)
33	*PTPRC*	−2.9	(−1.8)	−3.5
34	*JAK1*	−2.9	(−1.6)	−2.5
35	*USF1*	−3.3	−1.7	−2.8
36	*OAS1*	−3.3	(−1.5)	(−2.3)
37	*SLA2*	−3.4	(−1.3)	(−1.6)
38	*IL6ST*	−3.5	(−1.6)	−4.7
39	*MYC*	−3.6	(−2.1)	−2.1
40	*IFNG*	−4.1	−2.3	−2.0
41	*NR3C1*	−4.2	−2.4	−4.3
42	*SMAD2*	−4.2	(−1.7)	−2.5
43	*PPP2R1A*	−4.3	−2.5	−3.3
44	*IRF1*	−4.5	−2.1	−4.5
45	*GATA3*	−5.6	−2.5	−2.4
46	*STAT2*	−5.8	−2.2	−3.8
47	*IL10RA*	−6.4	−2.6	−4.5
48	*CSF1R*	−8.2	−2.9	−3.6
49	*SH2B1*	−8.7	−3.4	−5.2
50	*RPL13A*	−9.1	−3.3	−4.0
51	*SIT1*	−9.6	−3.0	−4.7
52	*STAT4*	−11.0	−3.6	−5.3
53	*A2M*	−11.3	−3.7	−3.8

Selected genes analyzed by JAK–STAT-related PCR array that showed statistically significant (p<0.05) up- or downregulation between the group 1 and the control group are shown. The magnitude of differences for groups 2 and 3 are also shown. Positive and negative numbers indicate up- and downregulation, respectively. Numbers in parentheses indicate a lack of statistical significance (p>0.05) in comparison with the control group.

Among the 14 genes that were downregulated more than 4-fold in group 1, 13 of them (*IFNG*, *NR3C1*, *PPP2R1A*, *IRF1*, *GATA3*, *STAT2*, *IL10RA*, *CSF1R*, *SH2B1*, *RPL13A*, *SIT1*, *STAT4*, and *A2M*) were also significantly downregulated in the groups 2 and 3. Only the downregulation of *SMAD2* in group 2 was not statistically significant (Table 2). Therefore, downregulation of these genes in MPN patients appears to be independent of *JAK2* V617F mutation.

### Re-analysis of *SOCS3* expression

**Figure 1:**
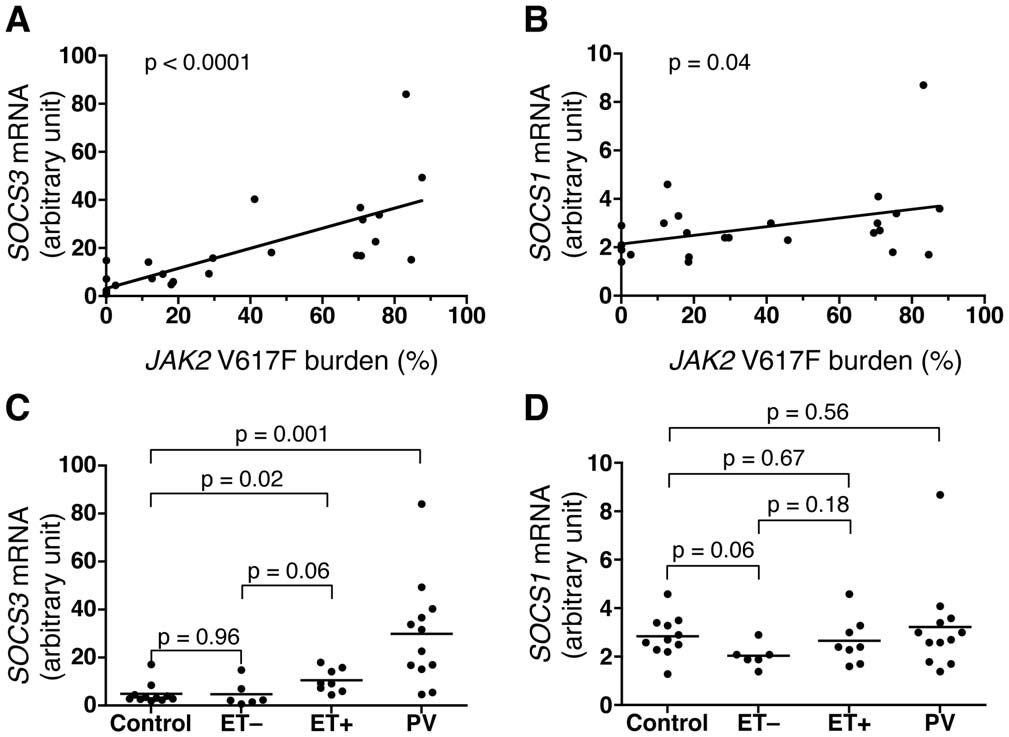
Expression of *SOCS3* and *SOCS1* mRNA in MPN patients. A. *SOCS3* mRNA levels in peripheral blood of MPN patients determined by qPCR (not PCR array) were calibrated with quantities of *HPRT1* mRNA and plotted against *JAK2* V617F mutation burden. The values are represented with an arbitrary unit. The line and p value for the slope were calculated based on a linear regression model. B. *SOCS1* mRNA levels plotted as in A. C. *SOCS3* mRNA levels were plotted by disease category. Control represents the healthy volunteers, and ET− and ET+ represent V617F-negative and -positive ET patients, respectively. PV represents PV patients. The p values were calculated by a t-test. D. *SOCS1* mRNA levels plotted as in C.

Elevated *SOCS3* mRNA expression in *JAK2* V617F-positive MPN patients was confirmed by individual quantitative PCR (qPCR) assay of peripheral blood cDNA samples from the 26 patients and 11 healthy volunteers. Expression levels of related *SOCS1* mRNA were also examined. As shown in Figure 1A, there was a clear correlation between *SOCS3* mRNA levels and *JAK2* V617F mutation burden, while only a marginal correlation was detected for *SOCS1* mRNA levels (Figure 1B). In these analyses, the control group was not included because of a lack of mutation data. The samples were then classified into 4 groups based on disease category and ET patients were divided into *JAK2* V617F-positive (ET+) and negative (ET−) groups. *SOCS3* expression in the PV and ET+ groups were significantly higher than those in the ET− and control groups (Figure 1C). The expression level of *SOCS3* was highest in PV patients and was moderately elevated in ET+ patients, while ET− patients showed the same level of expression as the control group. For *SOCS1* expression, there was no significant difference among the PV, ET+, and control groups (Figure 1D). However, ET− patients appeared to have lower *SOCS1* expression compared with the PV and control groups. This result is consistent with the qPCR array results showing a 4-fold reduction in group 3 and a 2-fold reduction in both group 1 and group 2 compared with the control group (Table 2).

### Re-analysis of *SPI1* expression

**Figure 2:**
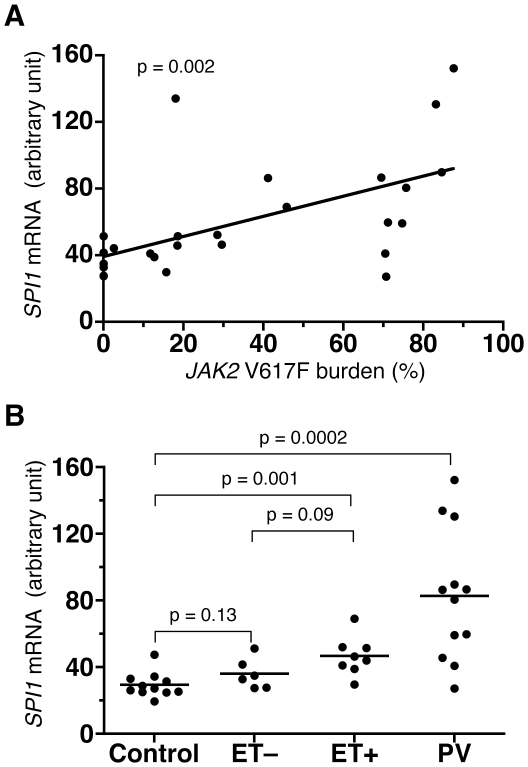
Expression of *SPI1* mRNA in MPN patients. A. *SPI1* mRNA levels in peripheral blood of MPN patients determined by qPCR (not PCR array) were calibrated with quantities of *HPRT1* mRNA and plotted against *JAK2* V617F mutation burden. The values are represented with an arbitrary unit. The line and p value for the slope were calculated based on a linear regression model. B. *SPI1* mRNA levels were plotted by disease category. Control represents the healthy volunteers, and ET− and ET+ represent V617F-negative and -positive ET patients, respectively. PV represents PV patients. The p values were calculated by a t-test.

In the PCR array experiment, compared with the control group, *SPI1* mRNA was upregulated 3.9-, 2.1-, and 1.6-fold among groups 1, 2, and 3, respectively (Table 2), suggesting a correlation between the *SPI1* expression level and the *JAK2* V617F mutation burden. This PCR array result was verified by individual qPCR assay (Figure 2A). PV and ET+ patients had significantly increased amounts of *SPI1* mRNA in peripheral blood compared to the control (Figure 2B).

### Analysis of signal transduction for *SPI1* expression

**Figure 3:**
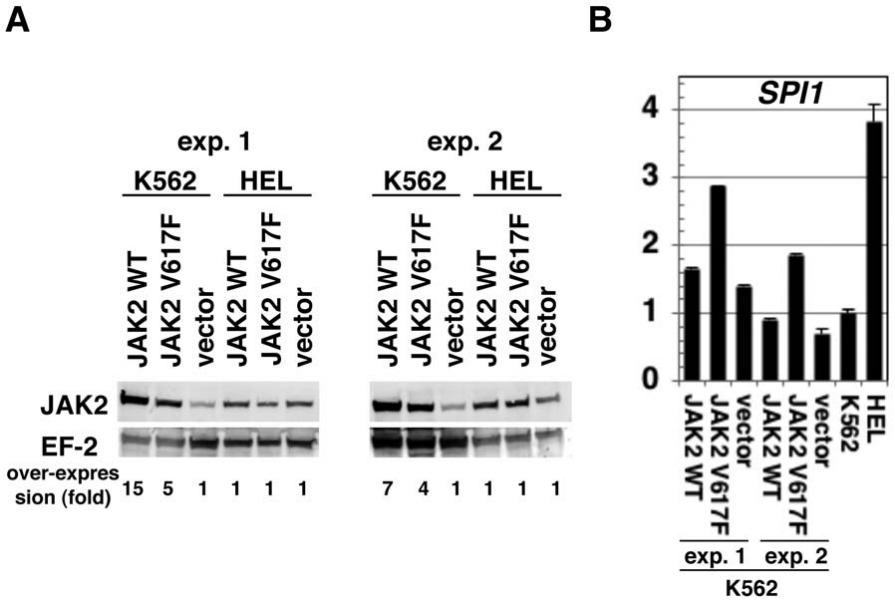
Induction of *SPI1* mRNA in K562 cells overexpressing V617F-type JAK2. A. Western blots showing the amounts of JAK2 protein inK562 and HEL cells infected with retrovirus vector encoding either wild-type (JAK2 WT) or V617F-type JAK2 (JAK2 V617F) or a mock vector (vector) and maintained in the presence of puromycin. The intensities of bands were calibrated with the band intensities of elongation factor 2 (EF-2) protein. Fold over-expression is shown below as the value for the mock infectant as 1. The results of two independent infections are shown under experiments (exp.) 1 and 2. Alexa 680-labeled secondary antibodies were used. B. *SPI1* mRNA levels in K562 cells prepared by the retroviral infection shown in A, along with those in non-infected K562 and HEL cells. The results were calibrated with 18S ribosomal RNA amount and represented with an arbitrary unit.

In zebrafish embryos, *spi1* expression is reported to be upregulated by a constitutively active *jak2a* mutant and reduced by suppression of *jak2a* by morpholino antisense oligonucleotide [11]. However, there are no reports on regulation of *SPI1* by JAK2 in humans. Hence, we set up an experiment using *in vitro* cultured cell lines to examine the role of JAK2 on the regulation of *SPI1*. K562 is a cell line derived from chronic myelogenous leukemia, which has a wild-type *JAK2* gene. HEL is a cell line derived from erythroid leukemia, which has a homozygous *JAK2* V617F mutation [12]. We retrovirally introduced wild-type and V617F-type *JAK2* transgenes and quantitated the expression levels of *SPI1* mRNA. The amount of JAK2 protein in the retroviral infectant increased in K562 cells but not in HEL cells (Figure 3A). As shown in Figure 3B, K562 cells overexpressing *JAK2* V617F exhibited 2-fold upregulation of *SPI1* mRNA compared with the mock transfectant. Such upregulation was not observed for wild-type *JAK2* transfectants. The reason for the lack of JAK2 overexpression in HEL cells is unclear, but it can be speculated that higher levels of JAK2 protein may be toxic to *JAK2* V617F-harboring cells.

**Figure 4:**
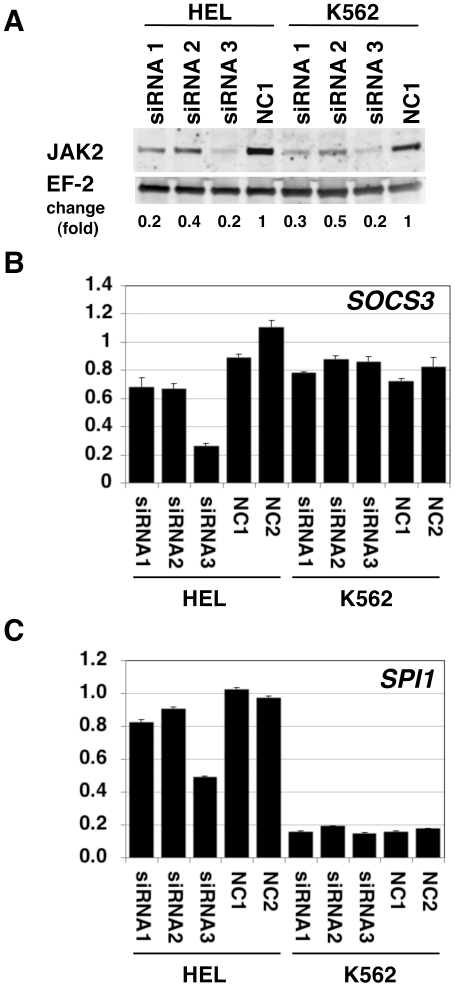
Reduction of *SOCS3* and *SPI1* mRNA in HEL cells transfected with JAK2 siRNAs. A. Western blots of JAK2 protein in HEL and K562 cells treated with siRNAs against JAK2 are shown. EF-2 was detected as a loading control. Cell lysates were prepared 24 h after siRNA transfection. Proteins derived from 1×10^5^ cells were loaded onto each lane. Alexa 680-labeled secondary antibodies were used. Three types of siRNA against JAK2 (siRNA1–3) and a negative control siRNA (NC1) are described in the Materials and Methods section. Fold change represents a ratio of band intensity of JAK2 and that of EF-2. B. *SOCS3* mRNA amount determined by qPCR. RNA was prepared 48 h after siRNA transfection. The values are expressed with an arbitrary unit as the mean of NC1 and NC2-treated HEL cells as 1. Error bars represent standard errors for triplicate measurements. C. *SPI1* mRNA amount shown as in B.

We then examined the effect of JAK2 downregulation by siRNA-mediated RNA interference (RNAi). Among the 3 types of commercially available siRNA tested against *JAK2*, one (siRNA3) was more effective than the other 2 in decreasing JAK2 protein expression in both HEL and K562 cells (Figure 4A). qPCR analysis revealed that *SOCS3* mRNA was downregulated by these siRNA with strongest inhibition for siRNA3 compared to negative controls in HEL cells (Figure 4B). However, this *SOCS3* mRNA suppression was not observed in K562 cells. This indicates that *SOCS3* expression in K562 cells is not driven by JAK2 signaling. When *SPI1* mRNA quantitation was similarly performed, the basal expression in HEL cells (control siRNA-transfected cells) appeared to be 6-fold higher than that in K562 cells (Figure 4C). In parallel with the *SOCS3* result, *SPI1* mRNA was downregulated by siRNA3 transfection only in HEL cells and not in K562 cells. High expression of *SPI1* mRNA and its inhibition by *JAK2*-siRNA transfection in HEL cells is consistent with the idea that *SPI1* gene is upregulated by V617F-type JAK2 in HEL cells.

**Figure 5:**
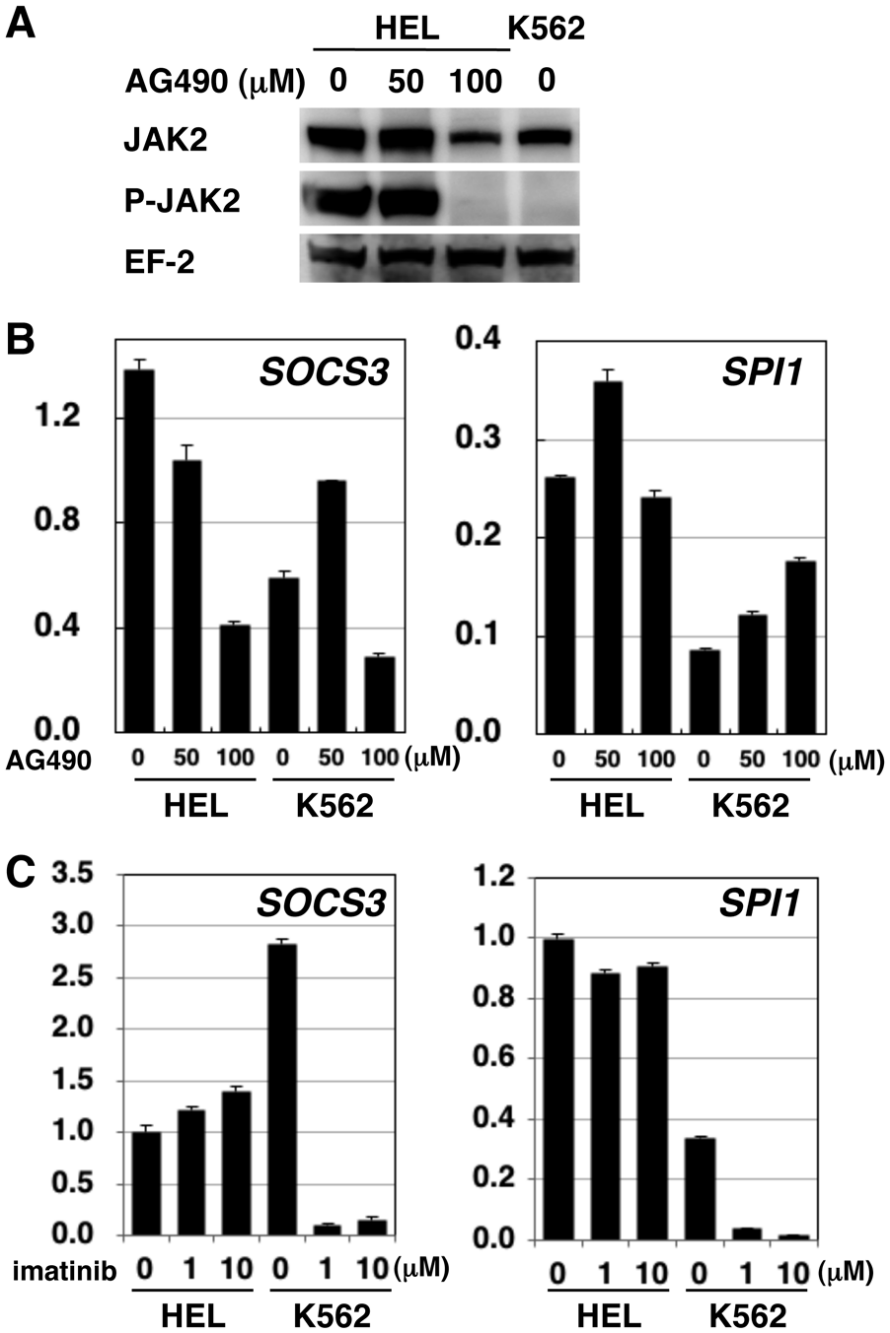
Effect of pharmacological inhibition of JAK2 and ABL1 kinases on *SOCS3* and *SPI1* expression. A. Western blots of total JAK2 and phosphorylated JAK2 (P-JAK2) are shown. EF-2 was detected as a loading control. Cell lysates were prepared 24 h after addition of indicated concentrations of AG490 to the culture medium. Proteins derived from 5×10^5^ cells were loaded onto each lane. Horseradish peroxidase-labeled secondary antibodies were used. B. *SOCS3* and *SPI1* mRNA amount determined by qPCR in cells 24 h after addition of indicated concentrations of AG490. The values are expressed with an arbitrary unit. Data at 48 h were similar (not shown). C. *SOCS3* and *SPI1* mRNA amount determined by qPCR in cells 48 h after addition of indicated concentrations of imatinib. The values are expressed with an arbitrary unit.

Finally, we examined the effect of a JAK2 inhibitor AG490 on *SPI1* and *SOCS3* expression. When the autophosphorylation of JAK2 at tyrosines 1007 and 1008 was examined using a phospho-JAK2-specific monoclonal antibody, HEL cells were found to contain phosphorylated JAK2, probably due to the kinase-activating V617F mutation. This phosphorylation was completely abrogated after culturing for 24 h in the presence of 100 µM AG490 (Figure 5A). The same JAK2 phosphorylation was undetectable in K562 cells even in the absence of AG490. Then, we examined the effects of AG490 on *SOCS3* and *SPI1* mRNA expression. As shown in Figure 5B, treatment of HEL cells with AG490 resulted in decreased expression of *SOCS3* mRNA but not *SPI1* mRNA. The response of K562 cells to AG490 was different from that of HEL cells. *SOCS3* mRNA was slightly enhanced at 50 µM but moderately suppressed at 100 µM. *SPI1* mRNA was slightly enhanced by AG490 in a dose-dependent manner.

Since JAK2 activates downstream genes through STAT3 or STAT5, and because both STAT3 and STAT5 are known to be activated by ABL1 signaling [13], [14], we tested an ABL1 inhibitor imatinib as well. When HEL cells were treated with imatinib, *SOCS3* mRNA slightly increased in a dose-dependent manner, while the *SPI1* mRNA level did not change (Figure 5C). In contrast, K562 cells treated with imatinib exhibited a significant reduction in the amounts of both *SOCS3* and *SPI1* mRNA, suggesting that the expression of *SOCS3* and *SPI1* is driven by ABL1 signaling in K562 cells.

Both AG490 and imatinib reduced proliferation of K562 cells but not HEL cells in a dose-dependent manner (Supplementary Figure S1). Therefore, we cannot formally exclude a possibility that suppression of *SOCS3* and *SPI1* expression by imatinib in K562 cells is attributed to a nonspecific cytotoxicity of imatinib.

## Discussion

In this study, we used quantitative allele-specific PCR to determine the *JAK2* V617F mutation burden in patients diagnosed with PV or ET and found a high and moderate mutation burden, respectively. These findings are consistent with previous reports [2], [7]. We also examined the expression profiles of 84 JAK–STAT-related genes using a commercially available PCR array and searched for genes that were differentially expressed among patient groups with different mutation burdens and/or clinical diagnoses. We found that *SOCS3* and *SPI1* expression were significantly elevated in *JAK2* V617F-positive patients.

SOCS3 belongs to the SOCS family of proteins that mediate negative-feedback inhibition of the JAK–STAT pathway [15]. SOCS3 expression is induced by erythropoietin, granulocyte-colony stimulating factor (G-CSF), interleukin-6 (IL-6), leukemia inhibitory factor, IL-23, and leptin. Ligand-induced dimerization of receptor tyrosine kinases activates JAK2 and subsequently STAT3, STAT5A, and STAT5B, which translocate to the nucleus and transactivate transcription of many target genes, including *SOCS3*. The induced SOCS3 protein in turn binds to phosphorylated tyrosine residues in the cytoplasmic tails of the same receptors that triggered SOCS3 induction and suppresses JAK2 activity both by direct binding to the JAK2 catalytic center and by promoting proteasomal degradation of JAK2.

Upregulation of *SOCS3* in the peripheral blood of *JAK2* V617F-positive MPN patients is consistent with a previous report [16] and is also expected, given existing information on the JAK–STAT signaling pathway [3]–[5]. In this study, we demonstrated that, unlike *SOCS1* mRNA, *SOCS3* mRNA expression level was clearly correlated with the *JAK2* V617F mutation burden and therefore, has a potential diagnostic value as a substitute for *JAK2* sequence analysis. Consistency between PCR array and individual qPCR assays for *SOCS3*, *SOCS1*, and *SPI1* provided us with a proof of the principle of the PCR array assay.

Our quantitation of *SOCS3* mRNA does not require fractionation of mononuclear cells or granulocytes in peripheral blood. Patient blood samples can be stored for up to 1 month at 4°C or for 3 days at ambient temperature once they have been mixed with the RNAlater solution for RNA stabilization. Our *SOCS3* mRNA quantitation procedure is also straightforward compared with the use of SOCS3 phosphorylation in sorted cell populations [17] and is suitable for use as a biomarker of JAK2 activation.

*SPI1* encodes the ETS family transcription factor PU.1, which is necessary for erythroid, myeloid, and lymphoid differentiation [6]. The target genes of PU.1 include those encoding IL-7 receptor α (IL-7Rα), macrophage colony stimulating factor receptor (M-CSFR), G-CSF receptor (G-CSFR), and granulocyte macrophage colony stimulating factor receptor α (GM-CSFRα). As ligand-engaged IL-7Rα recruits JAK1 and JAK3 and activates STAT5, PU.1 may activate STAT5 in developing lymphocytes. In developing erythrocytes, however, PU.1 deficiency does not affect the amount or phosphorylation status of STAT5 [18]. PU.1 is known to cooperate with STAT1 in promoter binding and transcriptional activation of the *FCGR1* gene encoding Fcγ receptor I [19]. PU.1 is also implicated in erythroleukemogenesis as PU.1 transgenic mice exhibit proliferation of proerythroblasts [20]. The reported link between PU.1 and cyclin-dependent kinase 6 (CDK6) may explain the oncogenic potential of this transcription factor [21].

It is noteworthy that other ETS transcription factors, such as. ERG, ETV1, ETV4, ETV6, FLI1, and FEV, are implicated in the pathogenesis of several cancers. For example, *ETV6* (also known as *TEL*) are occasionally fused with *RUNX1* (also known as *AML1*) in childhood precursor B-cell acute lymphoblastic leukemia [22]. Gene fusions found in prostate cancer often involve the *ERG*, *ETV1*, or *ETV4* gene [23]. Most Ewing sarcomas harbor gene fusions involving the *FLI1*, *ERG*, *ETV1*, *ETV4*, or *FEV* gene [24]. These reports suggest an oncogenic role of ETS transcription factors in general. Involvement of ETS transcription factors in PV is not surprising, given that its name is derived from that of the avian erythroblastosis virus, E26, which carries the *v-ets* (E Twenty-Six) oncogene [25].

**Figure 6:**
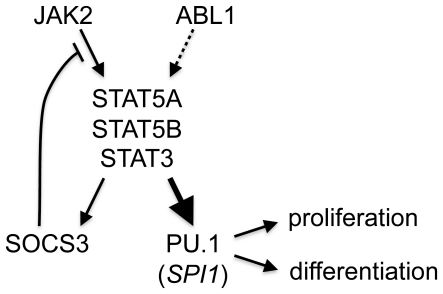
Proposed signaling pathways leading to *SPI1* and *SOCS3* gene expression. A thick arrow toward hematopoietic transcription factor PU.1 encoded by *SPI1* gene is a novel pathway reported in this study. Signaling from ABL1 to STAT5A, STAT5B, or STAT3 (dotted arrow) leading to SOCS3 and PU.1 expression is suggested by this study but its generalization in leukemic patients requires further validation. A negative feedback loop from JAK2 to STAT proteins and to SOCS3, which inhibits JAK2, was previously established. Enhanced expression of PU.1 may be involved in MPN development via its transcriptional control of genes regulating cellular proliferation and differentiation.

There have been extensive studies on gene regulatory elements for PU.1 expression. They have demonstrated that PU.1 is regulated by Oct-1, Sp1, GATA-1, SpiB, and PU.1 itself [6]. Notch1 also directly regulates PU.1 expression [26]. We observed increased *SPI1*/PU.1 expression in peripheral blood of our MPN patients. This increase was correlated with the *JAK2* V617F-mutation burden. Overexpression of the *JAK2* V617F mutant but not the wild-type *JAK2* in K562 cells also resulted in increased *SPI1* expression. RNAi against *JAK2* reduced *SPI1* expression in *JAK2* V617F-positive HEL cells. Therefore, our data are the first to suggest that *SPI1* expression is regulated by JAK2 in humans, possibly through STAT3, STAT5A, or STAT5B (Figure 6). Consistent with this hypothesis, upregulation of *SPI1*/PU.1 by GM-CSF, which also activates JAK2, in alveolar macrophages has been reported [27].

We found that *SPI1* was suppressed not only by RNAi against *JAK2* but also by pharmacological inhibition of ABL1. Since both JAK2 and ABL1 activate STAT3 and STAT5 [13], [14], *SPI1* expression may be mediated through STAT3 or STAT5. This speculation is consistent with reported reduction of *SPI1* expression after BCR-ABL1 inhibition by RNA interference or protein chaperon blockade in K562 [28], [29]. The lack of *SPI1* downregulation in AG490-treated HEL cells is difficult to interpret because of the possibility that the reduction of JAK2 signaling was insulated by attenuated negative feedback by SOCS3. In contrast to JAK2 inhibition by AG490, which affects the entire cell population, but may be incomplete, JAK2 knockdown by siRNA only affects the cells that incorporated siRNA. Considering the efficiency of siRNA transfection (approximately 60% of cells were positive 3 h after electroporation with fluorescence-labeled control siRNA) and overall reduction of JAK2 amount (approximately 80% for siRNA3, Figure 4A), suppression of JAK2 protein expression in individual cells may have progressed enough to prevent reversal by the weakened negative feedback by SOCS3. Refractoriness of downstream gene expression against upstream signal suppression by chemicals is a general trait of signaling pathways with negative feedback mechanisms like that of *SOCS3*. This point should be considered when pathway-targeted therapies are designed. In a study using zebrafish embryos where *spi1* was reduced by a *JAK2* antisense oligonucleotide [11], the effect of AG490 on *spi1* expression was not presented.

In this study, we did not analyze the genes downregulated in MPN patients in depth. Many of these downregulated genes were differentially expressed independent of *JAK2* mutations (Table 2), and further analysis of these genes may provide clues to the pathophysiology of MPN.

To summarize, examination of the expression profiles of 84 JAK–STAT-related genes in peripheral blood of MPN patients identified 2 upregulated genes. One is a known target, *SOCS3*, and the other is a potentially novel JAK–STAT target, *SPI1*, encoding the transcription factor PU.1. In addition to JAK2, ABL1 kinase may induce *SPI1* expression. Molecular analysis of patient samples such as those reported here will facilitate further understanding of hematological diseases and eventually lead to improved patient care.

## Supporting Information

Figure S1 A. Proliferation of HEL and K562 cells in the presence of indicated concentrations of AG490 subjected to expression analysis in Figures 5A and 5B was assessed by hemocytometer with trypan blue exclusion. Cell concentration is divided by the initial concentration of 4×10^5^ cell/ml and represented as the magnitude of change. B. Proliferation of HEL and K562 cells in the presence of imanitib subjected to expression analysis in Figure 5C was assessed by hemocytometer with trypan blue exclusion. Cell concentration is divided by the initial concentration of 2.5×10^5^ cell/ml and represented as the magnitude of change.
(TIF)

Table S1 **Patient List.** Summary of clinical data and *JAK2* V617F test for the 26 patients is shown. The normal range of each test is indicated in the brackets with female (F) or male (M)-specific ranges if available. Data are shown in 2 lines: upper is the data at the time of blood sampling for *JAK2* V617F mutation analysis and RNA preparation, and the lower within parentheses are those upon diagnosis. Treatment indicates administration of medication or bloodletting: HU for hydroxyurea, AP for allopurinol, AS for aspirin, and BL for bloodletting at the time of blood sampling. “No” treatments indicates that the patient was diagnosed freshly and had no previous history of treatment for MPN. Time from diagnosis to blood sampling for this study is indicated in months. Other abbreviations are: PV, polycythemia vera; ET, essential thrombocythemia; AS PCR, allele-specific PCR; WBC, white blood cell; RBC, red blood cell; Hb, hemoglobin; Ht, hematocrit; and PLT, platelet.
(XLS)

Table S2 **PCR primer sequence.** The primer sequences used in this study are shown.
(XLS)

Table S3 **PCR array data for analysis.** The PCR array data from 26 MPN patients (M plus number) and 11 healthy volunteers (N plus number) are shown. We analyzed this table using a web-based tool (RT2 Profiler) provided by SABiosciences.
(XLS)
