# Supplementary figures and images for: JAK2 V617F-Dependent Upregulation of PU.1 Expression in the Peripheral Blood of Myeloproliferative Neoplasm Patients
Source: PLoS One. 2011 Jul 18;6(7):e22148. doi: 10.1371/journal.pone.0022148 (PMC3138766; doi:10.1371/journal.pone.0022148)

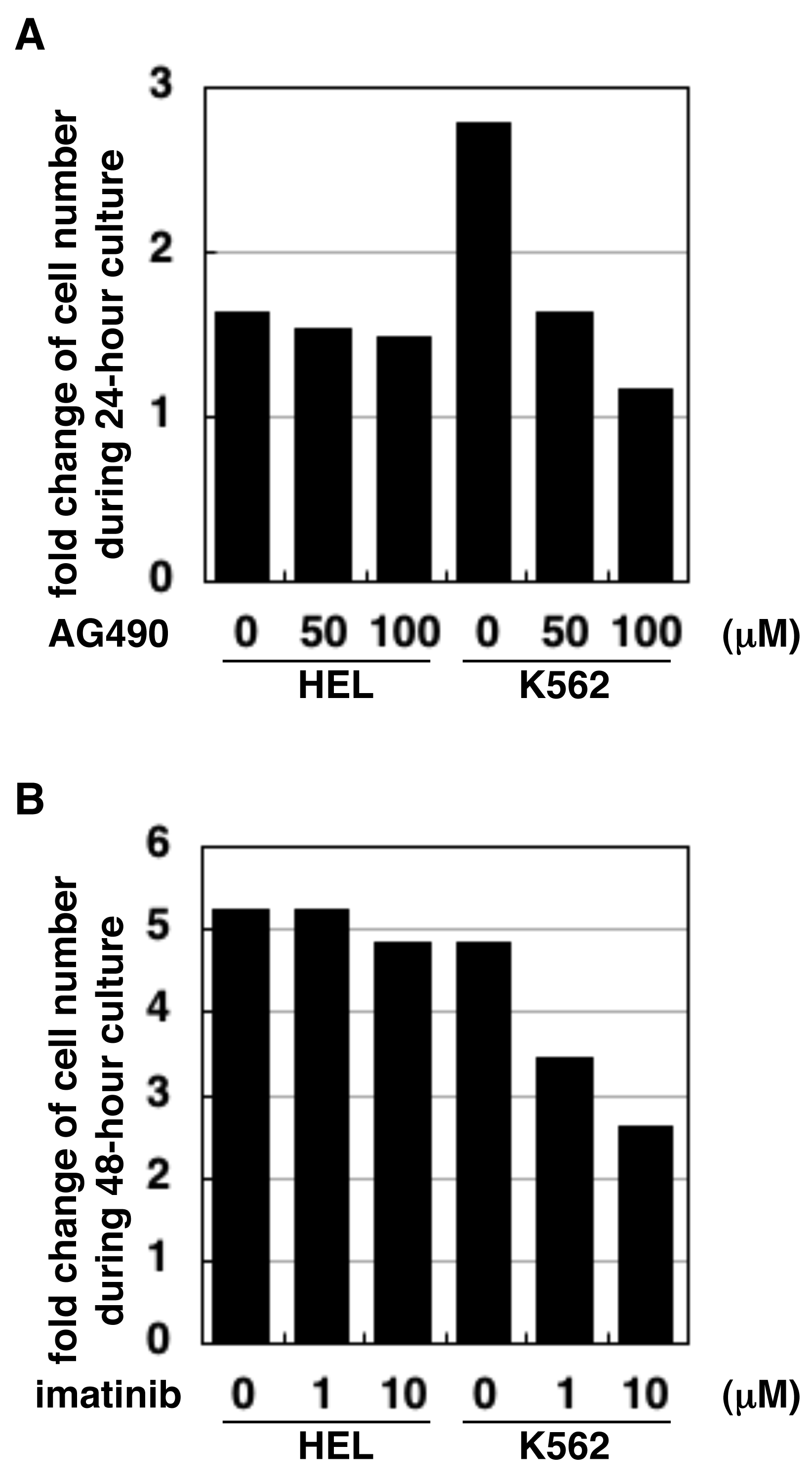

Supplement: Figure S1 — A. Proliferation of HEL and K562 cells in the presence of indicated concentrations of AG490 subjected to expression analysis in Figures 5A and 5B was assessed by hemocytometer with trypan blue exclusion. Cell concentration is divided by the initial concentration of 4×10^5^ cell/ml and represented as the magnitude of change. B. Proliferation of HEL and K562 cells in the presence of imanitib subjected to expression analysis in Figure 5C was assessed by hemocytometer with trypan blue exclusion. Cell concentration is divided by the initial concentration of 2.5×10^5^ cell/ml and represented as the magnitude of change. (TIF) [file pone.0022148.s001.tif]
